# An adaptive trial design for updating the threshold of a continuous biomarker

**DOI:** 10.1186/1745-6215-16-S2-P228

**Published:** 2015-11-16

**Authors:** Amy Spencer, Chris Harbron, Adrian Mander, James Wason, Ian Peers

**Affiliations:** 1 AstraZeneca Pharmaceuticals, Alderley Park, Cheshire, UK; 2 Roche Pharmaceuticals, Welwyn Garden City, UK; 3 MRC Biostatistics Unit Hub for Trials Methodology Research, Cambridge, UK

## 

Potential predictive biomarkers are often measured on a continuous scale, but in practice a threshold value to divide the patient population into biomarker “positive” and “negative” is disirable. Early phase clinical trials are increasingly using biomarkers for patient selection, but at this stage it is likely that little is known about the relationship between the biomarker and the treatment outcome. We describe a Phase II trial design with adaptive enrichment, which can increase power to demonstrate efficacy within a patient subpopulation, the parameters of which are also estimated. Our design enables us to learn about the biomarker and optimally adjust the threshold during the study, using a combination of generalised linear modelling an Bayesian prediction. At the final analysis, the hypothesis that “no population subset exists in which the novel treatment has a desirable effect” is tested. Through extensive simulations, we are able to show increased power over fixed threshold methods in many situations without increased false positive rates. We also show that estimates of the threshold which defines the population subset are unbiased and often more precise than those from fixed threshold studies. We provide an example of the method applied (retrospectively) to publically available data from a study of the use of tamoxifen after mastectomy by the German Breast Study Group, where progesterone receptor is the biomarker of interest.

